# 4-Hydr­oxy-2,2,6,6-tetra­methyl­piperidinium hydrogensulfate monohydrate

**DOI:** 10.1107/S1600536807067633

**Published:** 2008-01-04

**Authors:** Li Xiao, Yun-Hui Zhang, Ying Cui, Xing-Hua Jin, Wei Wang

**Affiliations:** a College of Pharmaceuticals and Biotechnology, Tianjin University, Tianjin 300072, People’s Republic of China; b School of Chemical Engineering, University of Science and Technology, Liaoning, Anshan 114051, People’s Republic of China

## Abstract

In the title compound, C_9_H_20_NO^+^·HO_4_S^−^·H_2_O, the piperi­dinium ring adopts a chair conformation. Inter­molecular O—H⋯O and N—H⋯O hydrogen bonds form an extensive three-dimensional network, which consolidates the crystal structure.

## Related literature

For useful applications of tetra­methyl­piperidinol, see: Gray (1991 ▶); Liu *et al.* (2006 ▶).
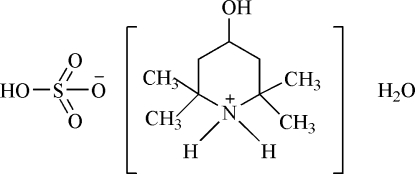


## Experimental

### 

#### Crystal data

C_9_H_20_NO^+^·HO_4_S^−^·H_2_O
*M* *_r_* = 273.34
Triclinic, 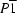

*a* = 8.334 (3) Å
*b* = 8.518 (3) Å
*c* = 10.245 (3) Å
α = 78.465 (5)°
β = 82.546 (5)°
γ = 71.586 (4)°
*V* = 674.3 (3) Å^3^
*Z* = 2
Mo *K*α radiation
μ = 0.26 mm^−1^
*T* = 294 (2) K
0.26 × 0.24 × 0.20 mm

#### Data collection

Bruker SMART CCD area-detector diffractometer
Absorption correction: multi-scan (*SADABS*; Bruker, 1997 ▶) *T*_min_ = 0.936, *T*_max_ = 0.951
3506 measured reflections
2374 independent reflections
1929 reflections with *I* > 2σ(*I*)
*R*_int_ = 0.016

#### Refinement

*R*[*F* ^2^ > 2σ(*F* ^2^)] = 0.040
*wR*(*F* ^2^) = 0.108
*S* = 1.06
2374 reflections
176 parameters
5 restraints
H atoms treated by a mixture of independent and constrained refinement
Δρ_max_ = 0.41 e Å^−3^
Δρ_min_ = −0.33 e Å^−3^

### 

Data collection: *SMART* (Bruker, 1997 ▶); cell refinement: *SAINT* (Bruker, 1997 ▶); data reduction: *SAINT*; program(s) used to solve structure: *SHELXS97* (Sheldrick, 1997 ▶); program(s) used to refine structure: *SHELXL97* (Sheldrick, 1997 ▶); molecular graphics: *SHELXTL* (Bruker, 1997 ▶); software used to prepare material for publication: *SHELXTL*.

## Supplementary Material

Crystal structure: contains datablocks global, I. DOI: 10.1107/S1600536807067633/cv2376sup1.cif

Structure factors: contains datablocks I. DOI: 10.1107/S1600536807067633/cv2376Isup2.hkl

Additional supplementary materials:  crystallographic information; 3D view; checkCIF report

## Figures and Tables

**Table 1:** Hydrogen-bond geometry (Å, °)

*D*—H⋯*A*	*D*—H	H⋯*A*	*D*⋯*A*	*D*—H⋯*A*
O2—H2⋯O1	0.83 (2)	1.76 (2)	2.576 (3)	168 (4)
N1—H1*A*⋯O5^i^	0.86 (2)	1.933 (17)	2.795 (3)	178 (2)
N1—H1*B*⋯O3^ii^	0.86 (2)	2.154 (19)	3.002 (3)	168 (2)
O6—H6*D*⋯O3^iii^	0.82 (2)	2.06 (2)	2.874 (3)	169 (4)
O6—H6*E*⋯O4^iv^	0.81 (2)	2.00 (2)	2.790 (3)	165 (4)

Symmetry codes: (i) 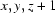; (ii) 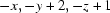; (iii) 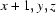; (iv) 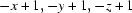.

**Fig. 1.**
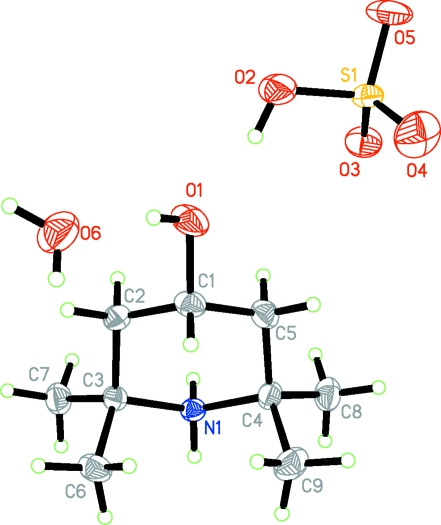
The content of asymmetric unit of (I) with the atomic numbering and 35% probability displacement ellipsoids.

## supplementary crystallographic information

### Comment

Tetramethylpiperidinol is an important intermediate used in the synthesis of hindered amine light stabilizer (HALS) (Gray, 1991; Liu *et al.*, 2006). The title compound, (I), is a new derivative of tetramethylpiperidinol. Herein we report its crystal structure.

In (I) (Fig. 1), the piperidinium ring adopts a chair conformation. The hydroxy group attached at C1 is in equatorial position. In the crystal, the intermolecular O—H···O and N—H···O hydrogen bonds (Table 1) form an extensive three-dimensional network, which consolidates the packing.

### Experimental

2,2,6,6-Tetramethylpiperidin-4-ol (40.0 g, 254 mmol) was dissolved in 98% H_2_SO_4_ (24.5 g) and then cooled to 278 K. With stirring, water (100 ml) was then added dropwise to the mixture over a period of 0.5 h. The mixture was stirred at 273–278 K for a further 3 h. The title compound (54.50 g) was obtained in powder form in a yield of 75.6%. Crystals of (I) were obtained by slow evaporation of a solution of water.

### Refinement

H atoms attached to atoms N and O were located in a difference map and refined with bond restraints O—H = 0.82 (2) Å, N—H = 0.86 (2) Å. C-bound H atoms were positioned geometrically (C—H 0.96–0.98 Å). All H atoms wrere refined as riding, with *U*_iso_(H)=1.2–1.5*U*_eq_ of the parent atom.

### Crystal data

**Table:**

C_9_H_20_NO^+^·HO_4_S^–^·H_2_O	*Z* = 2
*M**_r_* = 273.34	*F*_000_ = 296
Triclinic, *P*1	*D*_x_ = 1.346 Mg m^−^^3^
Hall symbol: -P 1	Mo *K*α radiation λ = 0.71073 Å
*a* = 8.334 (3) Å	Cell parameters from 1816 reflections
*b* = 8.518 (3) Å	θ = 2.6–26.2º
*c* = 10.245 (3) Å	µ = 0.26 mm^−^^1^
α = 78.465 (5)º	*T* = 294 (2) K
β = 82.546 (5)º	Plate, colourless
γ = 71.586 (4)º	0.26 × 0.24 × 0.20 mm
*V* = 674.3 (3) Å^3^	

### Data collection

**Table:**

Bruker SMART CCD area-detector diffractometer	2374 independent reflections
Radiation source: fine-focus sealed tube	1929 reflections with *I* > 2σ(*I*)
Monochromator: graphite	*R*_int_ = 0.016
*T* = 294(2) K	θ_max_ = 25.0º
φ and ω scans	θ_min_ = 2.0º
Absorption correction: multi-scan(SADABS; Bruker, 1997)	*h* = −9→6
*T*_min_ = 0.936, *T*_max_ = 0.951	*k* = −10→9
3506 measured reflections	*l* = −11→12

### Refinement

**Table:**

Refinement on *F*^2^	Secondary atom site location: difference Fourier map
Least-squares matrix: full	Hydrogen site location: inferred from neighbouring sites
*R*[*F*^2^ > 2σ(*F*^2^)] = 0.040	H atoms treated by a mixture of independent and constrained refinement
*wR*(*F*^2^) = 0.108	*w* = 1/[σ^2^(*F*_o_^2^) + (0.045*P*)^2^ + 0.4673*P*] where *P* = (*F*_o_^2^ + 2*F*_c_^2^)/3
*S* = 1.06	(Δ/σ)_max_ < 0.001
2374 reflections	Δρ_max_ = 0.41 e Å^−^^3^
176 parameters	Δρ_min_ = −0.33 e Å^−^^3^
5 restraints	Extinction correction: none
Primary atom site location: structure-invariant direct methods	

### Special details

**Table:**

Geometry. All e.s.d.'s (except the e.s.d. in the dihedral angle between two l.s. planes) are estimated using the full covariance matrix. The cell e.s.d.'s are taken into account individually in the estimation of e.s.d.'s in distances, angles and torsion angles; correlations between e.s.d.'s in cell parameters are only used when they are defined by crystal symmetry. An approximate (isotropic) treatment of cell e.s.d.'s is used for estimating e.s.d.'s involving l.s. planes.
Refinement. Refinement of *F*^2^ against ALL reflections. The weighted *R*-factor *wR* and goodness of fit *S* are based on *F*^2^, conventional *R*-factors *R* are based on *F*, with *F* set to zero for negative *F*^2^. The threshold expression of *F*^2^ > σ(*F*^2^) is used only for calculating *R*-factors(gt) *etc*. and is not relevant to the choice of reflections for refinement. *R*-factors based on *F*^2^ are statistically about twice as large as those based on *F*, and *R*- factors based on ALL data will be even larger.

### Fractional atomic coordinates and isotropic or equivalent isotropic displacement parameters (Å^2^)

**Table:**

	*x*	*y*	*z*	*U*_iso_*/*U*_eq_	
S1	0.14016 (7)	0.73866 (7)	0.24152 (5)	0.03302 (19)	
O1	0.4452 (2)	0.7411 (3)	0.46414 (17)	0.0535 (5)	
H1	0.547 (2)	0.720 (4)	0.457 (4)	0.080*	
O2	0.3063 (3)	0.7840 (3)	0.24406 (19)	0.0642 (6)	
H2	0.344 (5)	0.760 (5)	0.319 (2)	0.096*	
O3	0.0141 (2)	0.8333 (2)	0.33050 (17)	0.0468 (5)	
O4	0.1771 (3)	0.5626 (3)	0.2834 (2)	0.0752 (7)	
O5	0.0982 (3)	0.7967 (3)	0.10455 (17)	0.0676 (6)	
N1	0.1898 (2)	0.8477 (2)	0.83066 (18)	0.0273 (4)	
H1A	0.164 (3)	0.832 (3)	0.9156 (11)	0.033*	
H1B	0.124 (2)	0.9428 (18)	0.795 (2)	0.033*	
C1	0.3968 (3)	0.7257 (3)	0.6054 (2)	0.0361 (5)	
H1C	0.4668	0.6187	0.6519	0.043*	
C2	0.4211 (3)	0.8696 (3)	0.6595 (2)	0.0349 (5)	
H2A	0.3553	0.9749	0.6101	0.042*	
H2B	0.5396	0.8656	0.6444	0.042*	
C3	0.3682 (3)	0.8660 (3)	0.8082 (2)	0.0302 (5)	
C4	0.1464 (3)	0.7153 (3)	0.7725 (2)	0.0325 (5)	
C5	0.2122 (3)	0.7299 (3)	0.6255 (2)	0.0368 (5)	
H5A	0.1989	0.6383	0.5891	0.044*	
H5B	0.1442	0.8343	0.5763	0.044*	
C6	0.4904 (3)	0.7243 (3)	0.8968 (2)	0.0427 (6)	
H6A	0.4410	0.7137	0.9870	0.064*	
H6B	0.5952	0.7495	0.8946	0.064*	
H6C	0.5116	0.6208	0.8645	0.064*	
C7	0.3528 (3)	1.0332 (3)	0.8499 (3)	0.0435 (6)	
H7A	0.2729	1.1223	0.7972	0.065*	
H7B	0.4614	1.0530	0.8359	0.065*	
H7C	0.3145	1.0291	0.9427	0.065*	
C8	−0.0472 (3)	0.7621 (3)	0.7869 (3)	0.0463 (6)	
H8A	−0.0831	0.6808	0.7552	0.069*	
H8B	−0.0942	0.8711	0.7354	0.069*	
H8C	−0.0859	0.7639	0.8792	0.069*	
C9	0.2196 (4)	0.5397 (3)	0.8522 (3)	0.0497 (7)	
H9A	0.1695	0.4635	0.8278	0.075*	
H9B	0.1950	0.5431	0.9459	0.075*	
H9C	0.3401	0.5023	0.8328	0.075*	
O6	0.7835 (3)	0.6591 (3)	0.4742 (2)	0.0653 (6)	
H6D	0.858 (4)	0.702 (5)	0.442 (4)	0.098*	
H6E	0.814 (5)	0.591 (4)	0.540 (3)	0.098*	

### Atomic displacement parameters (Å^2^)

**Table:**

	*U*^11^	*U*^22^	*U*^33^	*U*^12^	*U*^13^	*U*^23^
S1	0.0324 (3)	0.0395 (3)	0.0236 (3)	−0.0090 (2)	0.0009 (2)	−0.0016 (2)
O1	0.0404 (10)	0.0952 (15)	0.0292 (9)	−0.0222 (11)	0.0056 (8)	−0.0224 (9)
O2	0.0442 (11)	0.1171 (19)	0.0365 (11)	−0.0394 (12)	0.0015 (8)	−0.0028 (11)
O3	0.0428 (10)	0.0516 (11)	0.0402 (10)	−0.0059 (8)	0.0048 (8)	−0.0131 (8)
O4	0.0928 (17)	0.0381 (11)	0.0867 (17)	−0.0123 (11)	−0.0044 (13)	−0.0057 (11)
O5	0.0535 (12)	0.1205 (19)	0.0234 (9)	−0.0260 (12)	−0.0040 (8)	0.0006 (10)
N1	0.0263 (10)	0.0300 (10)	0.0244 (9)	−0.0080 (8)	0.0000 (7)	−0.0038 (8)
C1	0.0352 (13)	0.0462 (14)	0.0251 (12)	−0.0095 (11)	0.0008 (9)	−0.0083 (10)
C2	0.0320 (12)	0.0450 (14)	0.0287 (12)	−0.0159 (10)	0.0019 (9)	−0.0040 (10)
C3	0.0272 (11)	0.0382 (12)	0.0264 (11)	−0.0122 (9)	−0.0018 (9)	−0.0043 (9)
C4	0.0349 (12)	0.0315 (12)	0.0345 (13)	−0.0155 (10)	−0.0001 (10)	−0.0055 (9)
C5	0.0376 (13)	0.0440 (14)	0.0328 (12)	−0.0144 (11)	−0.0019 (10)	−0.0123 (10)
C6	0.0321 (13)	0.0579 (16)	0.0334 (13)	−0.0081 (11)	−0.0067 (10)	−0.0030 (11)
C7	0.0448 (15)	0.0486 (15)	0.0456 (15)	−0.0221 (12)	−0.0031 (11)	−0.0139 (12)
C8	0.0398 (14)	0.0552 (16)	0.0521 (16)	−0.0243 (12)	0.0039 (12)	−0.0158 (13)
C9	0.0630 (18)	0.0324 (13)	0.0528 (16)	−0.0184 (12)	−0.0020 (13)	0.0003 (11)
O6	0.0456 (12)	0.0785 (16)	0.0653 (15)	−0.0238 (11)	0.0011 (10)	0.0083 (11)

### Geometric parameters (Å, °)

**Table:**

S1—O4	1.419 (2)	C4—C8	1.529 (3)
S1—O5	1.4411 (18)	C4—C9	1.530 (3)
S1—O3	1.4476 (18)	C4—C5	1.530 (3)
S1—O2	1.555 (2)	C5—H5A	0.9700
O1—C1	1.443 (3)	C5—H5B	0.9700
O1—H1	0.81 (2)	C6—H6A	0.9600
O2—H2	0.83 (2)	C6—H6B	0.9600
N1—C3	1.528 (3)	C6—H6C	0.9600
N1—C4	1.529 (3)	C7—H7A	0.9600
N1—H1A	0.86 (2)	C7—H7B	0.9600
N1—H1B	0.86 (2)	C7—H7C	0.9600
C1—C5	1.515 (3)	C8—H8A	0.9600
C1—C2	1.519 (3)	C8—H8B	0.9600
C1—H1C	0.9800	C8—H8C	0.9600
C2—C3	1.528 (3)	C9—H9A	0.9600
C2—H2A	0.9700	C9—H9B	0.9600
C2—H2B	0.9700	C9—H9C	0.9600
C3—C7	1.529 (3)	O6—H6D	0.82 (2)
C3—C6	1.531 (3)	O6—H6E	0.81 (2)
			
O4—S1—O5	114.56 (15)	N1—C4—C5	107.47 (17)
O4—S1—O3	112.68 (13)	C8—C4—C5	111.10 (19)
O5—S1—O3	111.13 (12)	C9—C4—C5	112.8 (2)
O4—S1—O2	107.34 (14)	C1—C5—C4	112.66 (18)
O5—S1—O2	103.30 (11)	C1—C5—H5A	109.1
O3—S1—O2	107.04 (12)	C4—C5—H5A	109.1
C1—O1—H1	106 (3)	C1—C5—H5B	109.1
S1—O2—H2	114 (3)	C4—C5—H5B	109.1
C3—N1—C4	120.80 (17)	H5A—C5—H5B	107.8
C3—N1—H1A	107.9 (16)	C3—C6—H6A	109.5
C4—N1—H1A	107.7 (16)	C3—C6—H6B	109.5
C3—N1—H1B	105.5 (16)	H6A—C6—H6B	109.5
C4—N1—H1B	105.6 (16)	C3—C6—H6C	109.5
H1A—N1—H1B	109 (2)	H6A—C6—H6C	109.5
O1—C1—C5	108.04 (18)	H6B—C6—H6C	109.5
O1—C1—C2	109.83 (19)	C3—C7—H7A	109.5
C5—C1—C2	110.27 (19)	C3—C7—H7B	109.5
O1—C1—H1C	109.6	H7A—C7—H7B	109.5
C5—C1—H1C	109.6	C3—C7—H7C	109.5
C2—C1—H1C	109.6	H7A—C7—H7C	109.5
C1—C2—C3	113.44 (18)	H7B—C7—H7C	109.5
C1—C2—H2A	108.9	C4—C8—H8A	109.5
C3—C2—H2A	108.9	C4—C8—H8B	109.5
C1—C2—H2B	108.9	H8A—C8—H8B	109.5
C3—C2—H2B	108.9	C4—C8—H8C	109.5
H2A—C2—H2B	107.7	H8A—C8—H8C	109.5
N1—C3—C2	107.19 (16)	H8B—C8—H8C	109.5
N1—C3—C7	105.65 (18)	C4—C9—H9A	109.5
C2—C3—C7	111.28 (19)	C4—C9—H9B	109.5
N1—C3—C6	110.75 (18)	H9A—C9—H9B	109.5
C2—C3—C6	112.80 (19)	C4—C9—H9C	109.5
C7—C3—C6	108.92 (19)	H9A—C9—H9C	109.5
N1—C4—C8	105.19 (18)	H9B—C9—H9C	109.5
N1—C4—C9	111.11 (19)	H6D—O6—H6E	111 (4)
C8—C4—C9	108.9 (2)		
			
O1—C1—C2—C3	178.44 (18)	C3—N1—C4—C8	−167.05 (19)
C5—C1—C2—C3	59.5 (2)	C3—N1—C4—C9	75.2 (2)
C4—N1—C3—C2	47.9 (2)	C3—N1—C4—C5	−48.6 (2)
C4—N1—C3—C7	166.70 (19)	O1—C1—C5—C4	−179.71 (19)
C4—N1—C3—C6	−75.5 (2)	C2—C1—C5—C4	−59.7 (3)
C1—C2—C3—N1	−50.8 (2)	N1—C4—C5—C1	51.7 (3)
C1—C2—C3—C7	−165.8 (2)	C8—C4—C5—C1	166.3 (2)
C1—C2—C3—C6	71.4 (2)	C9—C4—C5—C1	−71.1 (3)

### Hydrogen-bond geometry (Å, °)

**Table:**

*D*—H···*A*	*D*—H	H···*A*	*D*···*A*	*D*—H···*A*
O2—H2···O1	0.83 (2)	1.76 (2)	2.576 (3)	168 (4)
N1—H1A···O5^i^	0.86 (2)	1.933 (17)	2.795 (3)	178 (2)
N1—H1B···O3^ii^	0.86 (2)	2.154 (19)	3.002 (3)	168 (2)
O6—H6D···O3^iii^	0.82 (2)	2.06 (2)	2.874 (3)	169 (4)
O6—H6E···O4^iv^	0.81 (2)	2.00 (2)	2.790 (3)	165 (4)

Symmetry codes: (i) *x*, *y*, *z*+1; (ii) −*x*, −*y*+2, −*z*+1; (iii) *x*+1, *y*, *z*; (iv) −*x*+1, −*y*+1, −*z*+1.
